# H3K9 acetylation modification and TLR9 immune regulation mechanism in patients after anti-HBV treatment

**DOI:** 10.1097/MD.0000000000032431

**Published:** 2022-12-30

**Authors:** Haipeng Zhu, Ke Wang, Wei Du, Huanhuan Cao, Qingyang Zhong, Sichun Yin, Jianbo Zhong, Fawu Li

**Affiliations:** a Department of Infectious Diseases, the Dongguan People’s Hospital, Dongguan, Guangdong, P.R. China; b Department of Infectious Diseases, the Third Affiliated Hospital of Sun Yat-Sen University, Guangzhou, Guangdong, P.R. China.

**Keywords:** thymosin a1, entecavir, HBV, TLR9, immune regulation

## Abstract

To improve the curative effect of anti-hepatitis B virus (HBV) drugs, methods such as thymosin and entecavir combination have become a focus of clinical investigation. The aim of this retrospective experimental study was to explore the potential mechanism of action of thymosin a1 (Ta1) combined with entecavir in the treatment of HBV infection. A total of 28 patients with chronic hepatitis B, 29 patients treated with thymosin a1 and entecavir combination, and 15 healthy individuals were enrolled in this study. RT-qPCR was conducted to evaluate the mRNA levels of *TLR9* in peripheral blood mononuclear cells (PBMCs). The serum level of TLR9 protein was analyzed by ELISA. The binding of *TLR9* gene to the protein H3K9Ac in PBMCs was assessed by chromatin immunoprecipitation, and serum inflammatory factors were detected by Luminex technology. The expression levels of *TLR9* mRNA and serum *TLR9* protein in patients with HBV infection were significantly lower than those in subjects in the control group before treatment but increased after treatment with the Ta1 and entecavir combination. Moreover, the acetylation protein H3K9Ac was significantly bound to the promoter region of the *TLR9* gene in patients with HBV infection treated with the Ta1 and entecavir combination compared to that in patients with HBV infection without treatment. Furthermore, the expression levels of interleukin 6 (IL-6), interleukin 12 (IL-12), interferon gamma, and necrosis factor alpha in patients with HBV infection after the combination treatment were slightly decreased compared to those in patients with HBV infection without treatment. In conclusion, the histone acetylation modification of *TLR9* was significantly improved in patients with HBV infection after treatment with the Ta1 and entecavir combination, which elevated the expression of *TLR9* at the mRNA and protein levels and further regulated the expression of IL-6, IL-12, and other cytokines.

## 1. Introduction

China is a huge country with a high prevalence of liver disease.^[1–3]^ According to statistics, there are approximately 100 million people with chronic hepatitis B virus (HBV) infection in the country,^[4]^ with the rate of HBV infection being particularly high in Guangdong.^[5]^ Approximately 20% of infected patients may deteriorate to cirrhosis, liver cancer, or liver failure. Chronic hepatitis B has a complex pathogenesis.^[2]^ Currently available treatments primarily include antiviral therapy, immune regulation, antiinflammation and oxidation, and antifibrosis. In this context, entecavir, a potent and low-resistant anti-HBV drug, has emerged as a recognized treatment approach^[6]^; however, it has the drawbacks of limited efficacy, long course of treatment, and high relapse rate after drug withdrawal.^[7]^ From a clinical viewpoint, it is important to further improve its efficacy and shorten the course of treatment. A number of studies on HBV treatment approaches using thymosin a1 (Ta1) combined with entecavir have indicated that this treatment regimen can improve serum alanine aminotransferase recovery rate, HBV-DNA negative conversion rate, serum HBsAg negative conversion rate, and serum HBeAg/anti-HBe conversion rate.^[8–11]^ Nevertheless, it also exerts a few and slight clinical side effects.^[12]^ Entecavir exerts a direct antiviral effect primarily by inhibiting the activation of HBV polymerase, inhibiting the formation of negative strand by the reverse transcription of pregenomic mRNA, and inhibiting DNA-dependent DNA synthesis.^[13]^ Ta1 primarily regulates the immune function of the body against the virus.^[14,15]^ Therefore, it is extremely important to explore the antiviral mechanism of thymosin a1 combined with entecavir in the treatment of chronic hepatitis B.

Toll-like receptors (TLRs) are an important class of receptors involved in innate immunity and recognize foreign microorganisms, which can initiate the direct killing effect of innate immunity and mediate the secondary onset of adaptive immunity.^[16,17]^ *TLR9* is one of the receptors exerting multiple effects and a large influence.^[18]^ Ta1 can improve the antiinfective ability of the body by activating the TLR signaling pathway.

Previous studies have shown have found that Ta1 can upregulate *TLR9* expression via plasma cell-like DCs, inducing the expression of IL-10 and IL-12.^[19]^ Ta1 can also acquire antifungal innate immunity and protective Th1 cell immune response by activating signaling pathways such as those of *TLR2* and *TLR9*.^[20]^ It can also significantly enhance the ability of pDCs to secrete INF-α through the *TLR9* signaling pathway, clearing CMV.^[21]^ However, regarding anti-HBV infection treatment, there has been no relevant research on whether Ta1 influences *TLR9* expression.

Previously, when exploring the role of TLR1-10 in chronic hepatitis B, it was found that the expressions of only *TLR9* and *TLR10* were related to HBV load, suggesting that these 2 recognition receptors are closely related to HBV replication.^[22,23]^ The extracellular region of *TLR9* is crucial for identifying foreign microorganisms.^[24]^ It has the ability to recognize HBV virus and initiate immune response by identifying nonmethylated CpG gene sequences. Recent research has demonstrated that *TLR9* may be involved in the development of chronic hepatitis B.^[25]^ Xu et al found that patients with chronic hepatitis B have lower *TLR9* mRNA expression than normal individuals in peripheral blood mononuclear cells.^[26]^ HBV might inhibit the mRNA expression of *TLR9* in the peripheral blood mononuclear cells (PBMCs) of patients with chronic hepatitis B as a certain starter factor, causing immune escape or immune tolerance. This view was also confirmed by Vincent et al^[27]^ who found that in the pDCs of HBV-infected patients, HBV can downregulate the mRNA and protein expression of TLR9 to reduce the production of INF-α, which in turn causes persistent viral infection. For TLR9, there are traditional regulations and epigenetic modifications as well. Numerous studies have shown that the acetylation modification of histone H3 and lysine 9 (H3K9) is related to gene transcription development, whereas deacetylation is related to gene inactivation.^[28–30]^ Importantly, the literature reports that the state of histone acetylation modification is closely related to various viral infections. Our previous study compared the acetylation modification state of histone H3K9 of peripheral blood CD4^ + ^T cells in the whole genome promoter region under different disease states of chronic hepatitis B and found that H3K9 acetylation (H3K9Ac) modification regulation exists in 2 sequence regions of *TLR9*, suggesting that *H3K9* acetylation modification of *TLR9* also plays an essential role in the occurrence and development of chronic hepatitis B.^[22]^

In the current treatment of chronic hepatitis B based on thymosin and entecavir, emphasis is placed on whether *TLR9* affects the body’s immunity, whether HBV regulates *TLR9* expression, whether *TLR9* is related to the therapeutic effect, and the mechanism of action of *TLR9* in patients with hepatitis B, all of which will provide an important theoretical basis for determining the pathogenesis of chronic hepatitis B and the clinical antiviral mechanism. To continue our research on chronic hepatitis B,^[22,31,32]^ real-time PCR, Luminex, and chromatin immunoprecipitation technology were adopted to investigate the relationship among *TLR9* expression in PBMCs, secretion levels of the corresponding downstream inflammatory factors, and HBV load in patients with chronic hepatitis B receiving treatment with the Ta1 and entecavir combination from the aspects of clinical treatment and immune mechanism. It is confirmed that *TLR9* is involved in immune response in the anti-HBV process during treatment with the Ta1 and entecavir combination. The results of this study would provide a new basis for the antiviral immune control theory of chronic hepatitis B, which has social and clinical application significance.

## 2. Materials and Methods

### 2.1. Research objects and sample collection

The patients with chronic hepatitis B (serum HBsAg positive for more than 6 months) (18–65 years old) untreated or treated with Thymosin a1 (Ta1) combined with entecavir who were hospitalized in the Department of Infectious Diseases, Dongguan People’s Hospital from December 2017 to February 2019 were enrolled as research objects, and their general and clinical data were collected. They signed informed consent. Inclusion criteria: The diagnosis complies with “Guidelines for the Prevention and Treatment of Chronic Hepatitis B” jointly developed by Hepatology Branch of the Chinese Medical Association and Infectious Disease Branch of the Chinese Medical Association in 2015. It also refers to “Prevention and Treatment Program for Viral Hepatitis” jointly revised by Infectious Diseases and Parasitic Diseases Branch, Hepatology Brach of Chinese Medical Association in 2000. The patients with serum HBsAg-positive, HBeAg-positive and HBV DNA ≥ 2 × 10E4 IU/mL or HBeAg-negative and HBV DNA ≥ 2 × 10E3 IU/mL were included in this study.

Exclusion criteria: Patients with decompensated liver cirrhosis; those receiving other antiviral drugs and immunizations within 6 months; those with HAV, HCV, HDV, HEV, HIV, and other viral infections; patients with HCC or an AFP level of ≥ 400 ng/mL for more than 1 month; patients requiring immunosuppressive treatment or radiotherapy and chemotherapy due to other diseases; those with a positive pregnancy test or breastfeeding patients; and those who could not follow the study schedule or refused to sign informed consent were excluded from this study. As a midway withdrawal criterion, patients can withdraw from the study at any time before sample collection.

This study has been reviewed and approved by the Ethics Committee of Dongguan People’s Hospital (No. 2017079). Venous blood (5 mL) was collected from the cubital vein into an anticoagulant tube containing 500 µL of heparin. PBMCs were isolated within 2 hours after blood collection to detect *TLR9* mRNA expression. H3K9 acetylation was detected for the *TLR9* gene. Simultaneously, another blood sample (5 mL) was collected from the subject into an anticoagulation tube. It was centrifuged, and the separated plasma was collected and stored at −20°C for the detection of inflammatory factors.

### 2.2. Treatment regimens

A total of 28 patients with chronic hepatitis B (chronic hepatitis B [CHB] group), 29 patients treated with (thymosin a1 plus entecavir group), and 15 healthy volunteers (control group) were enrolled in this study. Patients in the thymosin a1 plus entecavir group were subcutaneously injected with thymosin a1 (1.6 mg/time, 2 times/week; Chengdu Diao Pharmaceutical Group) for 3 months plus oral entecavir (0.5 mg/day; Sino-American Shanghai Squibb Pharmaceutical Co., Ltd.) for 3 months. The 3 groups showed no significant differences in terms of gender and age (*P* > .05). HBV load was detected using the HBV-DNA quantitative detection kit from Daan Gene Co., Ltd., and HBsAg/HBeAg quantitative detection was performed using Abbott’s chemical fluorescence detection kit.

### 2.3. TLR9 mRNA expression during combination therapy

PBMCs isolated from blood samples were extracted with total RNA using the Trizol method (total RNA Extraction Kit, DP431, Tiangen), and the purity (NanoDrop2000, Thermo) was measured by an ultraviolet spectrophotometer. Reverse transcription was conducted following the reverse transcription box instructions (Fast King One Step RT-PCR Kit, KR123, Tiangen). Real-time PCR (Talent qPCR PreMix (SYBR Green), FP209, Tiangen) was performed using the designed *TLR9* gene-specific primers. The primers and amplification conditions were: F-5′-AACTGGCTGTTCCTGAAGTC-3′, *R*-5′-TGCCGTCCATGAATAGGAAG-3′, annealing temperature 55℃, product length 394 bp; internal reference gene β-actin: F-5′-AGGCCAACCGCGAAGATGACC-3′, *R*-5′-GAAGTCCAGGGCGACGTAGCAC-3′, annealing temperature 55℃, product length 350 bp. The Ct data was obtained by the Bio-Rad PRISM Sequence Detection software of the Fluorescence quantitative PCR instrument.

### 2.4. Detection of serum inflammatory factors by Luminex technology

Serum samples were isolated and preserved. The protein expression of 4 indicators, viz., interleukin 6 (IL-6), IL-12p70, interferon gamma (IFN-γ), and necrosis factor alpha (TNF-α), in human serum samples was detected using a multifactor detection antibody chip platform and R & D Systems High Sensitivity Cytokine Premixed Magnetic Luminex Performance Assay kit (FCSTM09-04). Regarding the experimental procedure, the sample was diluted 5 times with the same dilution as the dilution standard substance, that is 20 µL of sample stock solution was added to 80 µL of diluent.

All required reagents and standard substances were prepared as described in the kit instructions. The black microplate with a transparent bottom was removed from the dense kit equilibrated to room temperature. Unused strips were sealed with a sealing plate film. Standard substances and experimental samples of different concentrations were added to the wells, with a volume of 50 µL per well. The prepared microsphere working solution was resuspended and mixed before adding to each well, with a volume of 50 µL per well. After the reaction, the wells were sealed with a special sealing tape, and the plate was placed in a microplate shaker and incubated for 2 hours under 800-rpm shaking conditions at room temperature. Then, the reaction plate was placed in a handheld magnetic stand and held for 1 minute, after which the liquid in the plate was shaken off. Next, 100 µL of washing solution was added to each well, held for 1 minute, and then shaken off. There was no need to dry it on absorbent paper. The washing step was repeated twice, and the plate was washed 3 times.

Biotinylated detection antibody working solution (50 µL) was added to each well of the plate and sealed with a sealing tape, after which the plate was incubated in a microplate shaker for 1 hour at room temperature under 800-rpm shaking conditions. Next, streptavidin-PE working solution (50 µL) was added to each well and sealed with a sealing tape, after which the plate was incubated in a microplate shaker for 30 minutes at room temperature under 800-rpm shaking conditions. Then, washing solution (100 µL) was added to each well to resuspend the microspheres, and the plate was incubated in a microplate shaker for 2 minutes at room temperature under 800-rpm shaking conditions, followed by testing on a multifactor analyzer (Luminex 200) within 90 minutes. Based on the obtained fluorescence signal value and the concentration of each indicator substance, a five-parameter equation fitting was conducted using the software Xponent3.1, and the concentration of the unknown sample was calculated.

### 2.5. Chromatin immunoprecipitation and *H3K9* acetylation detection of *TLR9* gene

PBMCs in blood were separated from all fresh samples using PBMc separation solution, and the separation volume of each sample should be not less than 10 million cells. The predicted binding sites of *TLR9* gene and *H3K9* acetylation are as follows. >chr3: 52226200-52226513 (reverse complement)

AGCCCCTGGAGGGCAGAGAGCAGGGCAGGACAGCC AGAAGGGAGGTGGCACCTGCATGGG TCTAGGAGAGAGGATGGGGGCTGGTGGACA TGGGGACGGTGGGCTGTGGGCACACCCTTC AGCATGGTGGACCCAGCAGAACTTGCTGAG GGGCCCGGGGTCACCCAAGGCTTTGGGCCC GAGGAATCTGAGTCTCCTCACCTAGATCAG AAGGAGAGTGGGAAGAACTGATGGGGAGGA CTGGCTGGCGGCCCTGCTGGGCCAGCACAC ACCTGGCCTCTAGGAGCCCCATCTGGAGTG ACGTGGTGTGTGCT

>chr3: 52226753-52227102 (reverse complement)

TGTACATAATTCAGCAGATATCAAGCACTT ACTATGTGCTGGGCACTGTACTGGATCCTG GGGATGCAGATAAAAGATCACTGCCCTTA AGAAGCTGACATTCCAGCAGGGGAATAAGA CGATATACAATAAACCATGAAAGATCAATG ATCCGGTGTGCTAGCAGTTAAAAAATGTTA GGACAAAGAGAAACATAGACCAGGCAAAGG AGCTCAGGAGTGCCAGATCTGGGGTGGGAG GTTTGTAAGAAGGCTGGATGGCCCTGTTGA GAGGGTGACATGGGAGCAGAGACATAATGG AGGCAAAGGAGGGGTCATATGAGACTTGGG GGAGTTTTCAGGCAGAGGGAA

>chr3: 52227754-52228023 (reverse complement)

TTCACCCTCCCTGCCCCCACCACCTACCCC TGTCAAGATGGGTAACTTAATCACATCTGC GAAGTCGTTTTTGCCACACTGTGGGGTGTT GGGGTCACGTGTTTGCAGGTTTGGGGAATT AGGACAAGGATCTCTGAGAGGGACTTTATG CAGCCTCCCACATGGGATAAGGGCTCCTCC TCGAAGGCTTCCCAGCCTCCCTGGGCTGAG GCCAGGACAAGTTTTTCTGTGGACATCGAT ATCGGTGTCTCCAAG CTGAGT GTGTCCATG

A cell lysis buffer (100 µL) was added to the PBMCs isolated from the blood, and then the sample was vortexed, placed in an ice bath, and centrifuged, followed by removal of the supernatant. Next, 100 µL of MNase Digestion Buffer was resuspended and precipitated. Micrococcus nuclease (0.25 µL) was added with thorough mixing and water bath, and then 10 µL of MNase stop solution was added to stop the reaction, followed by placing in an ice bath, centrifugation, and removal of the supernatant. Subsequently, 50 µL of Lysis Buffer 2 was resuspended and precipitated, and then it was placed in an ice bath, vortexed, and centrifuged, followed by supernatant collection (the expression of H3K9 acetylation of the target protein in the sample was detected by the WB method).

Each supernatant (5 µL) sample obtained from the above-described steps was stored at −20°C as the input. Next, 45 µL of the supernatant was placed into a centrifuge tube containing IP dilution buffer. The positive control IP was 10 µL of anti-RNA polymerase II antibody, the negative control IP was 2 µL of normal rabbit IgG, and the target-specific IP was 5 µg of antibody. For each IP, 500 µL of diluted lysate was added to the plug spin column, and then the primary antibody was added for overnight incubation at 4°C. ChIP-Grade Protein A/G plus Agarose (20 µL) was added to each IP and incubated for 1 hour on a shaker, before discarding the supernatant by centrifugation. IP Wash Buffer (500 µL) was added before discarding the supernatant by centrifugation. The plug column was placed back into the 1.5 mL centrifuge tube, 150 µL of IP elution buffer was added to wash the resin, and then the resuspended magnetic beads were incubated. Next, 6 µL of 5 M NaCl and 2 µL of 20 mg/mL proteinase K were added and mixed thoroughly. For unfreeze input, 150 µL of IP elution buffer, 6 µL of 5 M NaCl, and 2 µL of 20 mg/mL proteinase K were added.

DNA binding buffer (750 µL) was added to a DNA clean-up column, followed by centrifugation to discard the supernatant. Next, the remaining sample was added to the same DNA clean-up column followed by the addition of 750 µL of DNA column wash buffer. Centrifugation was conducted twice to discard the supernatant. Subsequently, the DNA elution solution was added, followed by centrifugation to collect purified DNA, and then qPCR detection was performed. The primers used in this step are as follows: the translate system (20 µL) consisted of the forward primer, reverse primer, GoTaq^®^qPCR Master Mix, and GoTaq^®^qPCR Master Mix. The amplification system was predenatured at 95°C for 10 minutes, denatured at 95°C for 15 seconds, and extended at 60°C for 1 minutes. This process was repeated for 50 cycles of reaction. The Ct data were obtained using the Bio-Rad PRISM Sequence Detection software of the fluorescence quantitative PCR instrument.

### 2.6. Outcome measures

The primary outcome measures were the expression level of *TLR9* mRNA and serum *TLR9* protein and the binding of *TLR9* gene to the protein H3K9Ac in PBMCs. The secondary outcome measures were HBV-DNA load and expression levels of serum inflammatory factors.

### 2.7. Statistical analysis

Data were analyzed using the SPSS20.0 software (IBM Corp, Armonk, NY, TX). Normally distributed measurement results were expressed as mean ± standard deviation. Comparison between 2 groups was performed using Student *t* test. Comparison between more than 2 groups was performed using one-way analysis of variance. *P* < .05 indicated a statistically significant difference.

## 3. Results

### 3.1. Clinical sample information

General and serological data such as age and levels of alanine aminotransferase, total bilirubin, prothrombin activity, and HBV load of the enrolled subjects were collected (Table 1). There were no significant differences among 3 groups of samples in terms of gender, age, and BMI (*P* > .05), and the HBV load in the treatment group decreased significantly.^[22]^

**Table 1:** The clinical results between control, HBV and Ta1-ETV treatment group (*x* ± s).

Indicators	Control	CHB	Tal-ETV	*P* value
Patients (n)	15	28	29	–
Age^a^ (yr)	35.34 ± 7.65	39.71 ± 13.20	47.71 ± 13.20	.211
Gender (M/F)	12/3	19/9	21/8	.794
BMI (kg/m)	24.16 ± 5.33	22.19 ± 7.61	25.29 ± 5.66	.992
HBsAg (+)	0	28	29	.151
HBeAg (+)	0	16	9	.093
ALT^a^ (IU/L)	23.6 ± 2.9	986.5 ± 82.7	42.3 ± 6.6	.000
TBIL^a^ (µmol/L)	10.9 ± 2.1	197.1 ± 29.3	25.2 ± 3.9	.000
PTA^a^ (%)	86.1 ± 9.2	49.9 ± 5.8	81.2 ± 7.7	.019
HBV DNA^b^	ND	7.71 ± 1.1	0	.032

ALT = alanine aminotransferase, CHB = chronic hepatitis B, HBV = hepatitis B virus, PTA = prothrombin activity, Ta1 = thymosin a1, Ta1-ETV = thymosin a1 plus entecavir, TBIL = total bilirubin.

a For age and ALT, TBIL, PTA and HBV DNA (log10 IU/mL), mean SD is shown for each group.

b Log^10^ IU/mL.

### 3.2. Correlation between combined anti-HBV therapy of thymosin a1 and entecavir and TLR9 gene expression

**Figure 1.**
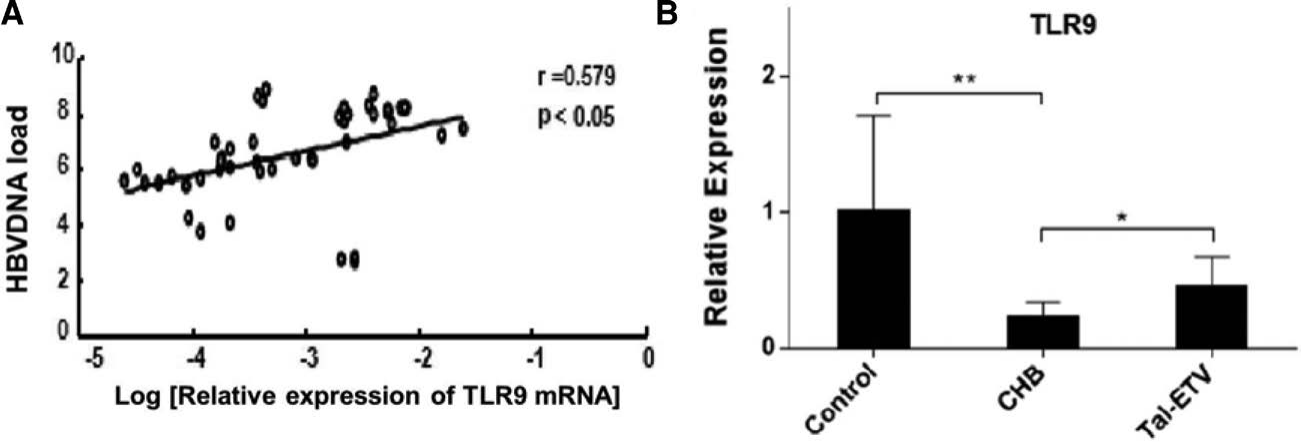
(A) Correlation between the expression of *TLR9* mRNA and serum HBV viral load in patients with chronic hepatitis. (B) Relative expression levels of *TLR9* mRNA in different populations. HBV = hepatitis B virus.

Before studying the differential expression of TLRs in the PBMCs of HBV-infected patients, we first examined the relationship between *TLR9* gene expression and HBV-DNA load by real-time quantitative PCR. The results are depicted in Figure 1A. A significant positive correlation was detected between the expression of serum *TLR9* mRNA in PBMCs and serum HBV-DNA load in patients with chronic hepatitis B (n = 45). To investigate the effect of *TLR9* expression on the pathogenesis of hepatitis B, we first explored the relative expression of *TLR9* mRNA in healthy volunteers, HBV-infected patients, and patients treated with entecavir and thymosin a1. The results are shown in Figure 1B.

**Figure 2.**
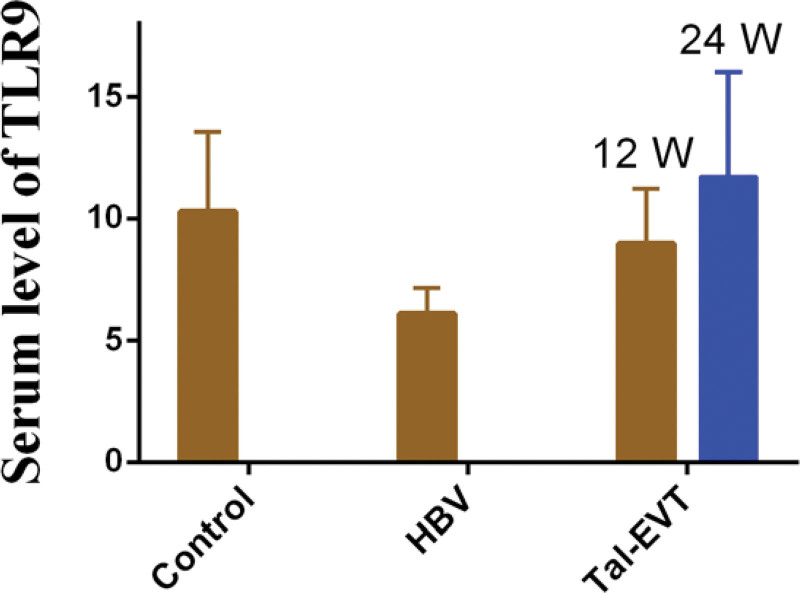
Comparison of serum *TLR9* protein expression levels in different populations.

To this end, we analyzed the expression of *TLR9* protein at different treatment time points by ELISA. As shown in Figure 2, compared with healthy individuals, patients with chronic hepatitis B had decreased serum *TLR9* protein expression under HBV stimulation, but the *TLR9* protein expression increased after a certain period of treatment. This result suggests that the combination of entecavir and thymosin a1 may affect the expression of TLR9 in patients with chronic hepatitis B.

### 3.3. H3K9 acetylation modification of TLR9 gene during anti-HBV treatment with thymosin a1 and entecavir

Using ChIP experiments, we examined the binding of the protein H3K9Ac to the promoter region of *TLR9* gene in the serum PBMCs of patients during the combined use of entecavir and thymosin a1. Table 2 shows the 3 predicted potential binding sites of *TLR9* gene and the protein H3K9Ac.

**Table 2:** The gene-specific primers of the binding sites for *H3K9Ac* and *TLR9*.

Gene	Binding sites	Primer	Primer sequence
*TLR9*	1	1F	5′-TGGACATGGGGACGGTG-3′
		1R	5′-AGCCAGTCCTCCCCATCAG-3′
*TLR9*	2	2F	5′-AAGAAGCTGACATTCCAGCAGG-3′
		2R	5′-TTACAAACCTCCCACCCCAG-3′
*TLR9*	3	3F	5′-TGTTGGGGTCACGTGTTTG-3′
		3R	5′-AGACACCGATATCGATGTCCAC-3′
*GAPDH* promoter	1	F	5′-CATGGGTGTGAACCATGAGA-3′
		R	5′-GTCTTCTGGGTGGCAGTGAT-3′

H3K9Ac = H3K9 acetylation.

**Figure 3.**
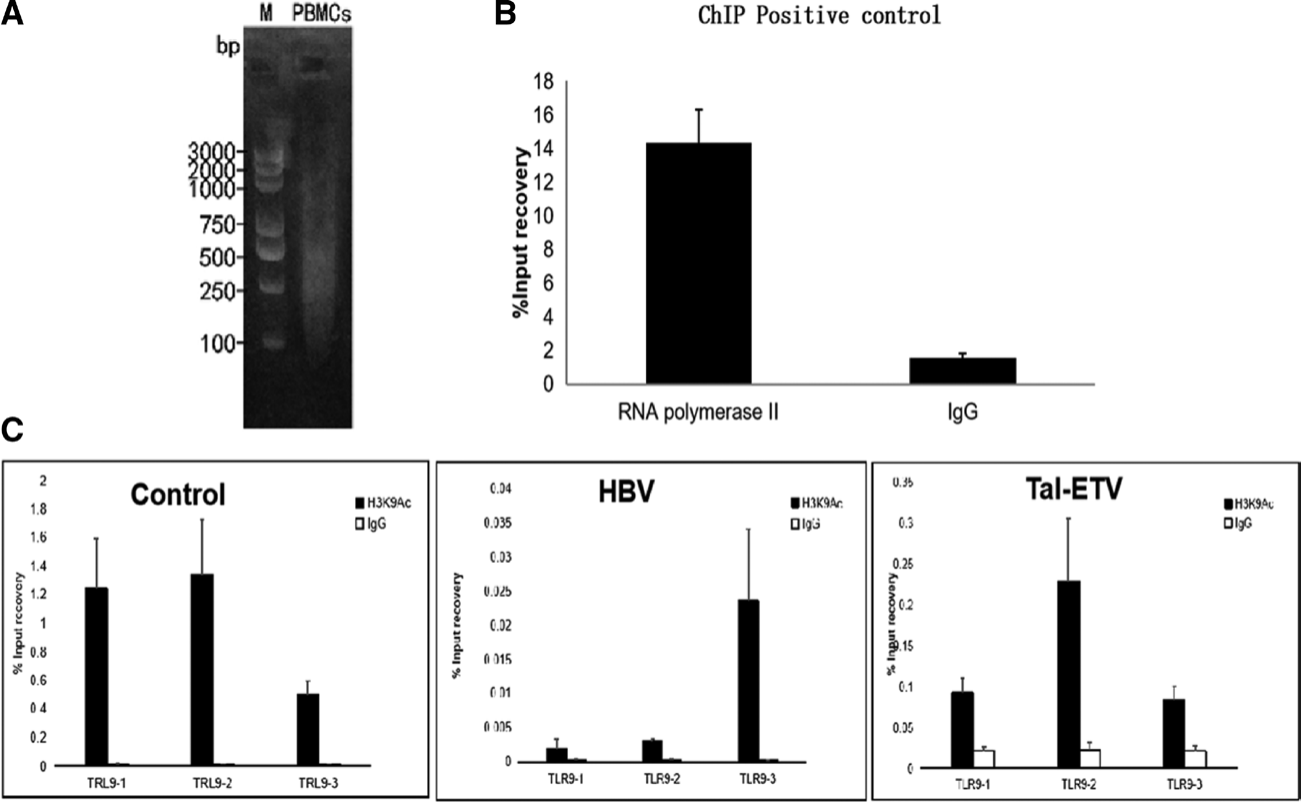
ChiP test to detect the binding of *TLR9* gene to the protein H3K9Ac in the serum PBMCs of different populations. (A) Fracture map of PBMCs chromatin extraction. (B) Positive control in the ChiP experiment. (C) Binding status of *TLR9* gene and the protein H3K9Ac in the PBMCs of different populations, ChiP = chromatin immunoprecipitation. H3K9Ac = H3K9 acetylation, PBMCs = peripheral blood mononuclear cells.

The gel electrophoresis stripes indicated that the DNA extracted from PBMCs was of high purity. Meanwhile, the target bands were clear and neat and could be amplified by PCR (Fig. 3A). RNA polymerase II was used as the positive control protein, and the gene sequence detected by qPCR was the *gapdh* promoter region (Fig. 3B). Rabbit IgG was used as the negative control protein, and the gene sequence detected by qPCR was the *gapdh* promoter region and target gene indicators. As shown in Figure 3C, *TLR9* gene binds with H3K9Ac in the serum PBMCs of healthy individuals, HBV-infected patients, and patients with chronic hepatitis B treated with entecavir and thymosin a1. The results revealed a change in the enrichment rate of H3K9Ac was disease-specific. Based on the detection of 3 sites, the differences in sites 2 and 3 were obvious between patients and normal individuals. The DNA sequence analysis also revealed that H3K9Ac exhibited the same change in the regional modification model as in other cell models. This is consistent with the finding that changes in histone modifications in fixed DNA sites induce the gene regulation model.

### 3.4. Detection of inflammatory factors during anti-HBV treatment with thymosin a1 and entecavir

**Figure 4.**
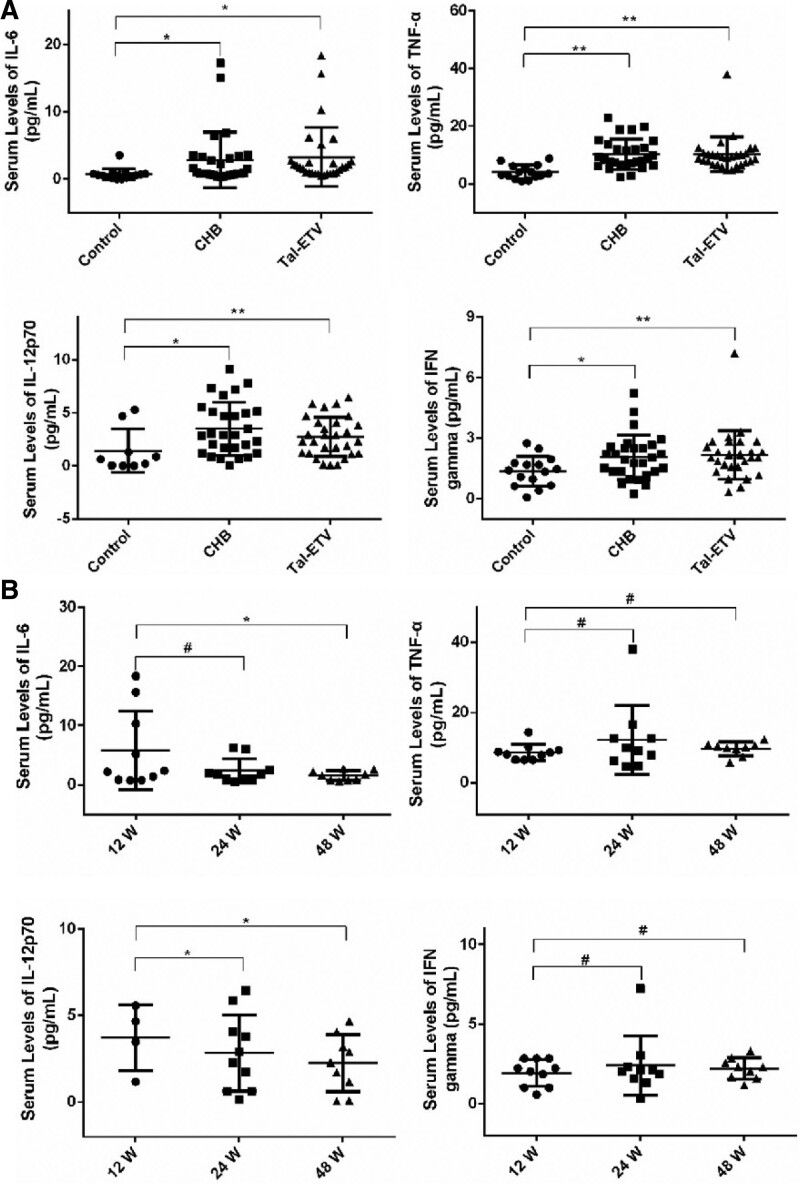
The serum levels of IL-6, IL-12, IFN-γ and TNF-α were determined by Luminex technology. (A) Mean (SD) serum levels of IL-6, IL-12, IFN-γ and TNF-α in patients with chronic hepatitis B (CHB) infection, those treated with the Tal-ETV combination for 24 weeks (24 W), and healthy control individuals. (B) Mean (SD) serum levels of IL-6, IL-12, IFN-γ and TNF-α in patients at different time points after Tal-ETV treatment. CHB = chronic hepatitis B, IFN-γ = interferon gamma, IL-12 = interleukin 12, IL-6 = interleukin 6, TNF-α = necrosis factor alpha.

To explore whether the H3K9 acetylation modification of *TLR9* gene was a consequence of immune regulation activation during application of the combination therapy, we first evaluated the expression levels of IL-6, IL-12, IFN-γ, and TNF-α in the serum of healthy volunteers, patients with hepatitis B, and patients treated with the combination therapy. As shown in Figure 4A compared with control group, the expression levels of IL-6, IL-12, IFN-γ and TNF-α were significantly increased in the CHB group. In addition, the levels of IL-6, IL-12, IFN-γ and TNF-α decreased slightly after combined treatment with entecavir and thymosin a1 compared with the CHB group. To examine the change in immune balance during the treatment of patients with chronic hepatitis B with entecavir and thymosin a1, we further analyzed the expression of immune factors such as IL-6, IL-12, IFN-γ, and TNF-α at the 12th, 24th, and 48th week of treatment cycles. As shown in Figure 4B. The expression levels of IL-6 and IL-12 decreased significantly, while the expression levels of IFN-γ and TNF-α increased slightly with the increase of the cycle of combination therapy. These results suggest that entecavir combined with thymotin a1 in the treatment of HBV can increase the expression of TLR9, and then regulate the expression of IL-6, IL-12 and other cytokines.

## 4. Discussion and conclusions

Current treatments for hepatitis B are primarily based on the use of antiviral drugs to improve inflammatory response and prevent viral infection of liver tissues, thereby improving clinical symptoms.^[4,5]^ However, there has been no perfect method for the antiviral treatment of hepatitis B. Problems such as poor curative effect, high recurrence rate, and long treatment course still plague frontline clinicians. To this end, it is of extraordinary positive significance for us to explore new treatment methods for patients with hepatitis B and clarify its mechanism of action, thereby improving the quality of life of these patients, improving the quality of medical care, and expanding the scope of medical services. Entecavir belongs to the class of 2-deoxyguanosine nucleoside analogs and is a clinically approved drug by the US FDA for the prevention and treatment of hepatitis B. It is primarily used to treat active viral replication and liver histology or as a serologically verified treatment. Thymosin a1 is a polypeptide substance in the primary active ingredient of thymosin, which can be used to improve cellular immune function. Thymosin a1 can help strengthen the antiinfective ability of the body, reducing the immune damage to liver cells, achieving repair effects, inhibiting liver cell apoptosis, and protecting the liver. Clinical studies have found that entecavir and thymosin a1 combination treatment for patients with chronic hepatitis B generally has a significantly better efficacy than entecavir-alone treatment, indicating that strengthening immune regulation during antiviral therapy can further strengthen the clearance effect of HBV, better improve patients’ clinical symptoms, and enhance the immunity of the body. Hence, exploring the molecular mechanism of action of entecavir and thymosin a1 in the treatment of chronic hepatitis B will provide an important theoretical reference for proposing additional treatment schemes.

A large number of studies have found that *TLR9* is activated in the immune cells of the body after stimulation by various viral infections, and it then exerts its immune function by stimulating the immune system.^[18,20]^ *TLR9* has the ability to identify HBV and initiate an immune response. *TLR9* expressed in cells is primarily located in the endoplasmic reticulum. Once infected viruses or bacteria are digested by intracellular lysosomes and endosomes, single-stranded DNA containing unmethylated CpG motifs are released, which can recruit *TLR9* for recognition and binding, thereby initiating the downstream immune activation response, inducing the expression of multiple inflammatory cytokines, and mediating the immune response. The genomic DNA of HBV is a circular partial double helix structure with a length of approximately 3200 bp. The methylation-specific software was adopted to identify nucleotide sequences containing CpG from the full-length HBV genome downloaded from NCBI. It was found that both B genotype and C genotype of the HBV genome contained a large number of CpG sites. Moreover, a CpG island was detected in the HBV genome; this region contains sites with a higher-than-the-usual frequency. During the replication or degradation of HBV, the presence of unmethylated CpG sequences in the open genome provides a theoretical basis for the recognition of *TLR9* and initiation of immune responses. Current studies have reported that *TLR9* expression is inhibited during the infection process in patients with chronic hepatitis B, causing immune escape.

In our previous research, in response to the differential expression of TLRs on PBMCs in HBV-infected patients, we found that the expression of *TLR9* mRNA on PBMCs in the serum of patients with chronic hepatitis B correlated significantly with the serum HBV-DNA load (Fig. 1A). To explore the effect of *TLR9* on the pathogenesis of hepatitis B, we further investigated the relative expression of *TLR9* mRNA in healthy volunteers, HBV-infected patients, and patients treated with the entecavir and thymosin a1 combination. As shown in Figure 1B, considering the expression level of *TLR9* in healthy volunteers as a control, the expression of *TLR9* in HBV-infected patients was significantly reduced, which was consistent with previous reports. HBV can downregulate the expression of *TLR9* mRNA and protein to reduce the production of immune factors. Nevertheless, the relative expression of *TLR9* mRNA increased significantly after the combination therapy, suggesting that HBV infection and *TLR9* treatment cause epigenetic changes. To this end, we explored the expression of *TLR9* protein by ELISA. We observed that compared with healthy individuals, patients with chronic hepatitis B had decreased *TLR9* protein expression in the serum under HBV stimulation, but the *TLR9* protein expression increased after a certain period of treatment. This indicates that *TLR9* plays an essential role in the treatment of chronic hepatitis B using entecavir and thymosin a1.^[24]^

During the treatment of chronic hepatitis B with the entecavir and thymosin a1 combination, the increase of *TLR9* mRNA levels may be achieved through 2 pathways, viz., mRNA transcription pathway and histone modification pathway. In the previous study, we performed a comparative analysis of the acetylation modification state of histone H3K9 in peripheral blood CD4^+^ T cells in the whole genome promoter region under different disease states of chronic hepatitis B, wherein we found that H3K9 acetylation modification regulation existed in 2 sequence regions of *TLR9* (Ch3: 52234647–52235096 and Chr3: 52234897–52235346), which suggests that the H3K9 acetylation modification of *TLR9* also plays an important role in the occurrence and development of chronic hepatitis B.^[22]^ To this end, we further conducted ChIP experiments to investigate the binding of the protein H3K9Ac to the *TLR9* gene promoter region in patients’ serum PBMCs during the combination therapy of entecavir and thymosin a1.^[28]^ As shown in Figure 3, the change in the enrichment rate of H3K9Ac was disease-specific and significant. In the detection of 3 loci, it was found that there were significant differences in loci 2 and 3 between patients and normal individuals. The DNA sequence analysis also revealed that H3K9Ac exhibited the same changes in the regional modification model as in other cell models, which is consistent with the finding that changes in histone modifications in fixed DNA sites induce the gene regulation model.

The pathogenesis of hepatitis B is associated with immune response and immune regulation disorders caused by HBV infection of liver cells. A large amount of interleukin and tumor necrosis factor α can appear in the serum of patients with hepatitis B. Interleukins can promote the differentiation and proliferation of *T* cells, B cells, NK cells, and other activated killer cells, forming a dynamic immune regulatory network to maintain the normal immune regulatory functions of the body.^[33]^ An imbalance of this network is closely related to the occurrence and development of various diseases. Using the liquid chip detection method, the expression levels of IL-6, IL-12, IFN-γ, and TNF-α in the serum of healthy volunteers, patients with hepatitis B, and patients undergoing combination therapy can be obtained, as shown in Figure 4. Compared with healthy individuals, the expression levels of IL-6, IL-12, IFN-γ, and TNF-α were significantly increased after HBV infection but decreased slightly after the combination therapy. In the different treatment cycles, the expression levels of IL-6 and IL-12 showed a significant downward trend, whereas those of IFN-γ and TNF-α showed a slightly upward trend, but the *P* value was nonsignificant. This may be due to the small sample size and a large number of confounding factors in the peripheral blood samples.

A strength of this retrospective experimental study is the inclusion of patients with CHB, patients with CHB after treatment, and healthy individuals. However, a previous study by Yan included only patients with CHB and those with CHB after treatment, without considering healthy individuals.^[34]^ Moreover, the data and research methods of this study are reliable and credible, which provides a theoretical basis for follow-up studies. There are also some potential limitations in our study. First, the sample size was relatively small, which must be increased in further studies. Second, this was a single-center study, and hence a multicenter study is needed in the future to support our results.

In conclusion, this study demonstrated that the H3K9 acetylation modification of *TLR9* is considerably important for patients with chronic hepatitis B.^[22]^ During the combination therapy with entecavir and thymosin a1, the histone acetylation modification of *TLR9* was significantly improved, which increased the expression of *TLR9* at the mRNA and protein levels and further regulated the expression of IL-6, IL-12, and other cytokines. The expression results correlated with the antiviral efficacy. These data confirm that *TLR9* participates in immune response in the anti-HBV process during treatment with the Ta1 and entecavir combination, which provides a new basis for the antiviral immune control of chronic hepatitis B and has social and clinical significance.

## Acknowledgements

We thank all the patients and healthy individuals for their participation.

## Author contributions

**Conceptualization:** Haipeng Zhu, Ke Wang.

**Data curation:** Wei Du, Huanhuan Cao.

**Formal analysis:** Jianbo Zhong.

**Investigation:** Qingyang Zhong, Fawu Li.

**Methodology:** Ke Wang, Wei Du, Huanhuan Cao.

**Statistical analysis:** Sichun Yin, Jianbo Zhong.

**Supervision:** Haipeng Zhu.

**Validation:** Huanhuan Cao.

**Writing – review & editing:** Haipeng Zhu.

**Writing – original draft:** Ke Wang.

## References

[1] Wang FS Fan JG Zhang Z. The global burden of liver disease: the major impact of China. Hepatology. 2014;60:2099–108. 25164003 10.1002/hep.27406 PMC4867229

[2] Xie YD Zhao CQ Wang JP. Alcohol consumption analysis among patients with liver disease in China. Chin Med J. 2019;132:420–30. 30707167 10.1097/CM9.0000000000000067 PMC6595713

[3] Wang LJ Zhu MY Cao LH. Liver stiffness measurement can reflect the active liver necroinflammation in population with chronicliver disease: a real-world evidence study. J Clin Transl Hepatol. 2019;7:313–21. 31915600 10.14218/JCTH.2019.00040 PMC6943212

[4] Chen Y Yang JE Tang JM. Predictive value of plasmacytoid dendritic cells and Toll-like receptor-9 regarding the treatment efficacy of interferon-α in HBeAg-positive chronic hepatitis B patients. Exp Ther Med. 2019;18:4541–6. 31798696 10.3892/etm.2019.8161 PMC6878902

[5] Tan AT Schreiber S. Adoptive T-cell therapy for HBV-associated HCC and HBV infection. Antiviral Res. 2020;176:104748. 32087191 10.1016/j.antiviral.2020.104748

[6] Liu Y Yao WM Si LL. Chinese herbal extract Su-duxing had potent inhibitory effects on both wild-type and entecavir-resistant hepatitis B virus (HBV) in vitro and effectively suppressed HBV replication in mouse model. Antiviral Res. 2018;155:39–47. 29702120 10.1016/j.antiviral.2018.04.017

[7] Terry CFY Vincent WSW Henry LYC. Tenofovir is associated with lower risk of hepatocellular carcinoma than entecavir in patients with chronic HBV infection in China. Gastroenterology. 2020;158:215–25. 31574268 10.1053/j.gastro.2019.09.025

[8] Wu BF Yang JY Zhang YL. 781 a clinical trial on anti-HBV-DC vaccine combined with lamivudine and thymosin-a1 in the HBeAg positive patients of chronic hepatitis B virus infection. J Hepatol. 2013;58:s318.

[9] Chan H. A meta-analysis of thymosin-a1 treatment in chronic hepatitis B virus (HBV) infection. J Hepatol. 2001;34:133.

[10] Saruc M Yuceya H Kucukmetin N. Comparison of interteron α2β monotherapy with the combination of thymosin αα1 and interferon α2b in the treatment of anti-HBe-postive chronic hepatitis B in turkey. Gastroenterology. 2001;120:A380–A380. 12063993

[11] Zhuang L You J Zhang B. Preliminary results of Thymosin-a1 versus interferon-α treatment in patients with HBeAg negative and serum HBV DNA positive chronic hepatitis B. World J Gastroenterol. 2001;7:407–10. 11819800 10.3748/wjg.v7.i3.407 PMC4688732

[12] Gregory BW Eugenia FR Minter H. Aimmunochemical and physical-chemical evidence for the presence of thymosin alpha1-peptide in dialyzable leukocyte extracts1. Immunobiol Transf Factor. 1983:395–411.

[13] Block TM Guo HT Guo JT. Molecular virology of hepatitis B virus for clinicians. Clin Liver Dis. 2007;11:685–706, vii. 17981225 10.1016/j.cld.2007.08.002 PMC2144742

[14] Wang FW Xu CY Peng RH. Effect of a C-end rule modification on antitumor activity of thymosin α1. Biochimie. 2018;154:99–106. 30096371 10.1016/j.biochi.2018.08.001

[15] Hannappel E Huff T. The thymosins: prothymosin α, parathymosin, and β-thymosins: structure and function. Vitam Horm. 2003;66:257–96. 12852257 10.1016/s0083-6729(03)01007-0

[16] Katherine AF Jonathan CK. Toll-like receptors and the control of immunity. Cell. 2010;180:1044–66. 10.1016/j.cell.2020.02.041 PMC9358771 32164908

[17] Bourquin C Pommier A Hotz C. Harnessing the immune system to fight cancer with Toll-like receptor and RIG-I-like receptor agonists. Pharmacol Res. 2020;154:104192. 30836160 10.1016/j.phrs.2019.03.001

[18] Qin CJ Sun JX He Y. Diurnal rhythm and pathogens induced expression of toll-like receptor 9 (TLR9) in Pelteobagrus vachellii. Fish Shellfish Immunol. 2019;87:879–85. 30794932 10.1016/j.fsi.2019.02.038

[19] Romani L Bistoni F Perruccio K. Thymosin alpha1 activates dendritic cell tryptophan catabolism and establishes a regulatory environment for balance of inflammation and tolerance. Blood. 2006;108:2265–74. 16741252 10.1182/blood-2006-02-004762

[20] Makita Y Suzuki H Kano T. TLR9 activation induces aberrant IgA glycosylation via APRIL- and IL-6–mediated pathways in IgA nephropathy. Kidney Int. 2020;97:340–9. 31748116 10.1016/j.kint.2019.08.022 PMC7372907

[21] Han NN Zhang Z Jv HY. Culture supernatants of oral cancer cells induce impaired IFN-α production of pDCs partly through the down-regulation of TLR-9 expression. Arch Oral Biol. 2018;93:141–8. 29913322 10.1016/j.archoralbio.2018.06.006

[22] Jin L Wang K Liu H. Genomewide histone H3 lysine 9 acetylation profiling in CD4+ T cells revealed endoplasmic reticulum stress deficiency in patients with acute-on-chronic liver failure. Scand J Immunol. 2015;82:452–9. 26173605 10.1111/sji.12341

[23] Shahrakyvahed A Sanchooli J Sanadgol N. TLR9: an important molecule in the fight against hepatitis B virus. Postgrad Med J. 2014;90:396–401. 24942353 10.1136/postgradmedj-2013-132309

[24] Martha RB Carlos A Felipe A. Humoral immune response and TLR9 gene expression in Pacific red snapper (Lutjanus peru) experimentally exposed to Aeromonas veronii. Fish Shellfish Immunol. 2015;42:289–96. 25462554 10.1016/j.fsi.2014.11.002

[25] Xie Q Shen HC Jia NN. Patients with chronic hepatitis B infection display deficiency of plasmacytoid dendritic cells with reduced expression of TLR9. Microbes Infect. 2009;11:515–23. 19289178 10.1016/j.micinf.2009.02.008

[26] Xu YF Hu YW Shi BS. HBsAg inhibits TLR9-mediated activation and IFN-α production in plasmacytoid dendritic cells. Mol Immunol. 2009;46:2640–6. 19501403 10.1016/j.molimm.2009.04.031

[27] Lo AO Wong VW Wong G. Mo1532 predictors of response to entecavir in chronic hepatitis B patients with incomplete virologic response to telbivudine in a four-year prospective cohort. Gastroenterology. 2012;142:S988–988.

[28] Lin T Sun L Lee JE. Changes of histone H3 lysine 23 acetylation and methylation in porcine somatic cells, oocytes and preimplantation embryos. Theriogenology. 2020;148:162–73. 32182524 10.1016/j.theriogenology.2020.03.006

[29] Li X Chen XR Zhou WJ. Effect of melatonin on neuronal differentiation requires CBP/p300-mediated acetylation of histone H3 lysine 14. Neuroscience. 2017;36419:45–59. 10.1016/j.neuroscience.2017.07.064 28782640

[30] Gebremedhin KG Rademacher DJ. Histone H3 acetylation in the postmortem Parkinson’s disease primary motor cortex. Neurosci Lett. 2016;6273:121–5. 10.1016/j.neulet.2016.05.060 PMC5067161 27241718

[31] Zhu HP Gu YR Zhang GL. Depression in patients with chronic hepatitis B and cirrhosis is closely associated with the severity of liver cirrhosis. Exp Ther Med. 2016;12:405–9. 27347069 10.3892/etm.2016.3271 PMC4906933

[32] Peng L Xie DY Lin BL. Autologous bone marrow mesenchymal stem cell transplantation in liver failure patients caused by hepatitis B: short-term and long-term outcomes. Hepatology. 2011;54:820–8. 21608000 10.1002/hep.24434

[33] Wang HL Luo H Wan X. Wenting Tan1, TNF-α/IFN-γ profile of HBV-specific CD4 *T* cells is associated with liver damage and viral clearance in chronic HBV infection. J Hepatol. 2020;72:45–56. 31499130 10.1016/j.jhep.2019.08.024

[34] Yan H Zhang X Lv Y. The effect of entecavir therapy on immune status in chronic hepatitis B patients. Iran J Immunol. 2019;16:84–91. 30864558 10.22034/IJI.2019.39409

